# Sex and race differences in the association of albumin with cognitive function in older adults

**DOI:** 10.1002/brb3.3435

**Published:** 2024-02-26

**Authors:** Yang Hu, Duo Lin, Min Song, Dongmei Wu, Yuqing Zhang, Gongbo Li, Haiyan Luo

**Affiliations:** ^1^ Department of Neurology The Second Affiliated Hospital of Chongqing Medical University Chongqing China; ^2^ Department of Neurology Zhongshan Hospital of Traditional Chinese Medicine Guangdong China

**Keywords:** albumin, Alzheimer's disease, cognitive impairment, dementia, older adults

## Abstract

**Background:**

With the increasing aging population, dementia has become a significant socioeconomic burden. However, the effects of albumin on delayed recall (DR) impairment remain unclear, and there are limited reports on sex and race differences in this relationship. This study aimed to investigate the association between albumin levels and DR impairment in older adults.

**Methods:**

A total of 1507 normal cognitive function and 553 DR impairment from the National Health and Nutrition Examination Survey (NHANES) 2011–2014 were included in this cross‐sectional analysis. Participants aged 60 years and above were assessed using the Consortium to Establish a Registry for Alzheimer's Disease DR (CERAD‐DR) test to evaluate cognitive function. Participants were categorized into DR impairment and normal cognitive function groups according to their CERAD‐DR scores. Logistic regression analyses, generalized additive models, and fitted smoothing curves were utilized for data analysis.

**Results:**

After adjusting for potential confounders, a negative association was found between albumin levels and cognitive function (odds ratio [OR] = 0.60, 95% confidence interval [CI] 0.41–0.87). Subgroup analysis stratified by sex, race/ethnicity, and age revealed that the negative association remained significant in men (OR = 0.53, 95%CI 032–0.87), Blacks (OR = 0.35, 95%CI 0.17–0.74), and the age group of 60–70 years (OR = 0.48, 95%CI 0.28–0.81). However, no significant association was observed in women (OR = 0.72, 95%CI 0.41–1.28), whites (OR = 0.58, 95%CI 0.31–1.07), or Mexican Americans (OR = 1.11, 95%CI 0.35–3.46), as well as the age group of 71–80 years (OR = 0.62, 95%CI 0.37–1.03).

**Conclusions:**

Our study suggests that elevated albumin levels are associated with a decreased incidence of cognitive function impairment, particularly in older men and Blacks. This finding indicates that maintaining high levels of albumin may be beneficial for cognitive function in older adults.

## 1 INTRODUCTION

Cognitive skills, including learning, memory, language, visual and spatial skills, and executive functions, play a crucial role in daily life (Daviglus et al., 2010). Cognitive dysfunction, which refers to impairment in cognitive abilities, can significantly impact patients’ daily functioning, leading to a burden on patients and their families (Daviglus et al., 2010). Cognitive impairment is prevalent in the elderly population, likely attributed to age‐related changes in the brain and cumulative damage over time (Rosano et al., 2016). Dementia, a severe form of cognitive impairment, affects approximately 6% of the population over 65 years old and up to 40%–70% of those over 95 years old (Qiu et al., 2007). With the global population aging, the number of people with dementia is projected to increase from 57.4 million cases in 2019 to 152.8 million cases by 2050 (GBD 2019 Dementia Forecasting Collaborators, 2022). In the United States, an estimated 8.7 million individuals aged 71 years or older are affected by dementia, making cognitive dysfunction a significant public health problem (Plassman et al., 2008). Moreover, the process from early cognitive decline to dementia is progressive and irreversible, with no effective treatment currently available. However, research suggests that early interventions and timely diagnosis can potentially delay the onset of dementia by even 1 year, leading to a 10% decline in dementia prevalence worldwide (Liu et al., 2020). Therefore, addressing cognitive dysfunction in the elderly is of utmost importance to mitigate the impact of this growing public health concern.

Serum albumin, the most abundant protein in human plasma, serves multiple biological functions and is primarily synthesized in the liver. It acts as a carrier protein, osmotic regulator, and antioxidant in the plasma, playing a crucial role in overall health status, aging, and neurodegeneration. As a carrier, serum albumin facilitates the stable presence and transport of hydrophobic and hydrophilic molecules, whereas its free sulfhydryl group (–SH) confers antioxidant properties to combat oxidative stress. Serum albumin has also been explored as a potential blood marker for malnutrition. A community‐based study of 2550 elderly Chinese individuals revealed that low albumin levels were independently associated with poorer cognitive function (Ng et al., 2008). Moreover, several population‐based studies have suggested that low albumin may be an independent risk factor for poor cognitive function and dementia (Zuccalà et al., 2005). However, two other studies reported there was no significant difference in cognitive function among different albumin levels group (Dik et al., 2005; Ravaglia et al., 2005).

Evidence is limited and controversial to the association between albumin and cognitive function. The association between serum albumin and delayed recall (DR) impairment has not been assessed before. Here, we used these representative samples of National Health and Nutrition Examination Survey (NHANES) 2011–2014 to evaluate the association between serum albumin and DR impairment in older adults. It has potential practical significance for preventing cognitive impairment and improving cognitive functioning in the elderly.

## 2 METHODS

### 2.1 Data collection and study population

**FIGURE 1:**
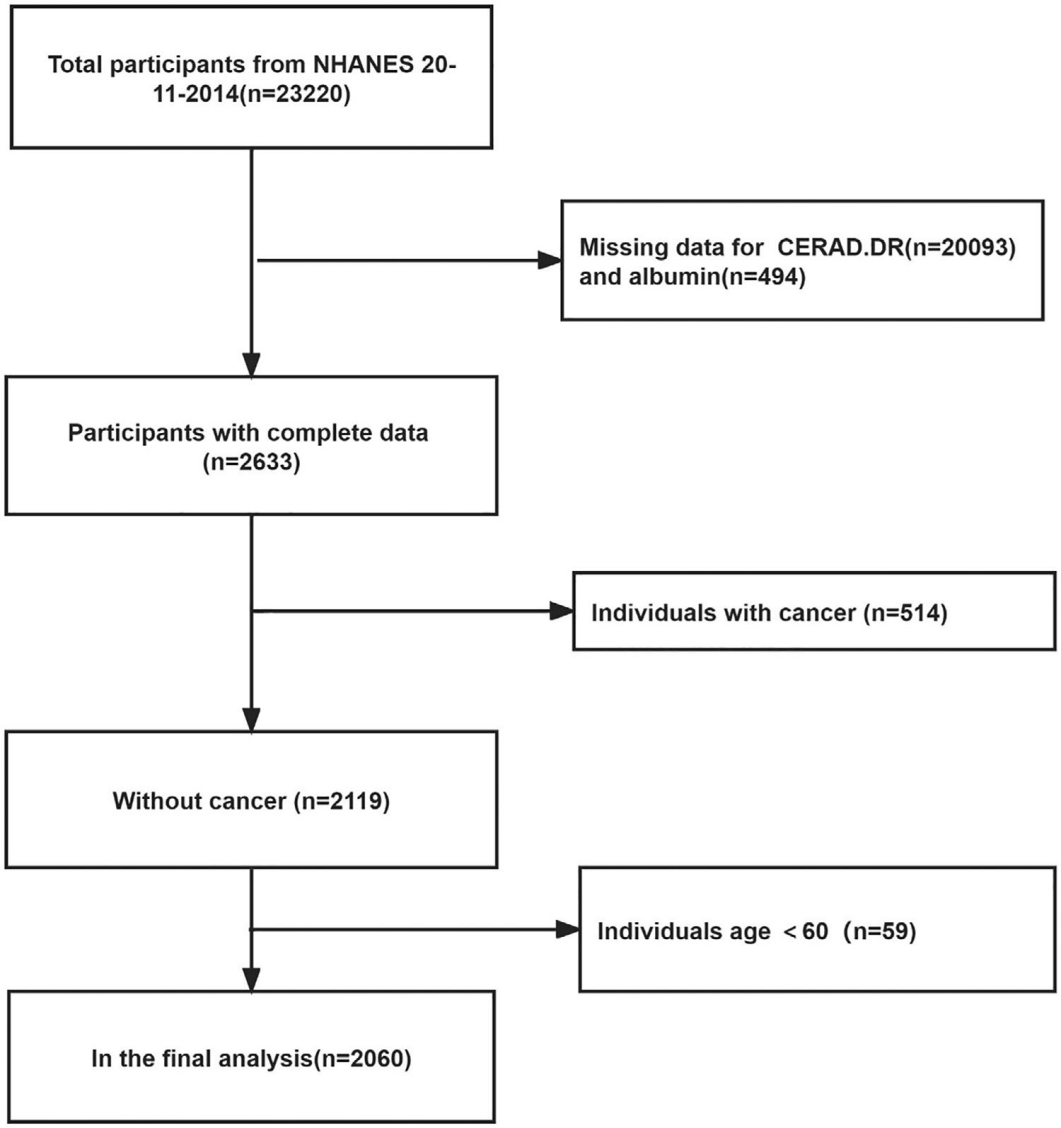
Flow chart of sample selection from the National Health and Nutrition Examination Survey (NHANES) 2011–2014.

The NHANES is a nationally representative series of surveys conducted in the United States since the early 1960s, aimed at investigating various health topics in the population. In this study, data from NHANES 2011 to 2014 were analyzed to evaluate cognitive function and serum albumin levels in participants aged 60 years and older. The study was approved by the National Center of Health Statistics (NCHS) Ethics Review Board, and written informed consent was obtained from all participants. Cognitive function was assessed using the Consortium to Establish a Registry for Alzheimer's Disease DR (CERAD‐DR) test. After excluding 20,093 participants with incomplete CERAD‐DR assessments, 494 with missing albumin data, 514 with cancer, and 59 participants whose age is less than 60. Cancer patients were excluded due to the potential influence of cancer‐related malnutrition, which is known to affect up to 80% of patients with advanced cancer (Tisdale, 2002). Cancer may have a significant influence on albumin. Finally, our analysis included 2060 participants. The selection process is shown in Figure 1.

### 2.2 Cognitive performance

The CERAD is a comprehensive test used to assess immediate and delayed learning ability for new verbal information (Morris et al., 1989). The CERAD word learning subtest (CERAD W‐L) involves participants reading aloud 10 random words, one at a time, and then trying to recall as many words as possible after presentation. Each of the 3 learning trials presents the 10 words in a different order, with a maximum score of 10 for each trial. The CERAD‐DR test is administered after completion of all cognitive performance tests and requires participants to recall the 10 unrelated words used during the first CERAD W‐L trial, approximately 8–10 min after the start of the W‐L trials. Cutoffs of <5 for CERAD‐DR have been previously used in the literature to distinguish potential cognitive impairment (Sotaniemi et al., 2012). DR ability is closely related to storage and retrieval in the memory process and can reflect memory function to some extent. Therefore, this index can be clinically selected to evaluate memory function.

### 2.3 Albumin and globulin

Serum albumin (g/dL) and serum globulin (g/dL) were measured with Beckman UniCel DxC800 Synchron instruments (Fan et al., 2020).

### 2.4 Covariates

Demographic variables, including age, sex, race/ethnicity, education, income‐poverty ratio (IPR), and body mass index (BMI), were self‐reported via questionnaire in a population‐based study. Lifestyle factors, such as smoking behavior and alcohol consumption, were also assessed. Biomarkers in blood, including alanine aminotransferase (ALT), aspartate aminotransferase (AST), and globulin, were measured. Health factors, including hypertension, diabetes, and stroke, were also recorded. Race/ethnicity was categorized into Mexican American, non‐Hispanic white, non‐Hispanic black, and other races. The DxC800 utilized kinetic rate and enzymatic rate methods to measure ALT and AST activity in serum or plasma, respectively. Education levels were defined as below high school, high school, and above high school. Height and weight measurements were collected by trained health technicians to calculate BMI. Smoking behavior was categorized as smokers and nonsmokers based on whether individuals smoked more than 100 cigarettes in their lifetime. Alcohol consumption was categorized as more than 12 glasses in 1 year and less than 12 glasses in 1 year. Poverty was defined using the IPR index, with cut‐points of <1.99, 1.99–3.49, and ≥3.5 (Vieux et al., 2019). Data on diabetes, hypertension, and cancer were obtained from the subjects through self‐reporting of their medical history.

### 2.5 Statistical analysis

Continuous values were presented as mean ± SD, whereas categorical values were expressed as count (%). The unpaired *t*‐test and *χ* ^2^ test were used to compare two groups of participants. Multivariate logistic regression models were employed to investigate the association between albumin levels and cognitive function. Participants were categorized into quartiles based on their albumin levels in the logistic regression analysis. Odds ratios (ORs) and 95% confidence intervals (CIs) for cognitive impairment were calculated for quartile 2, quartile 3, and quartile 4 (Q4) relative to quartile 1 (Q1). Three different logistic regression models were constructed: model 1 without any covariates, model 2 adjusting for age, sex, and race/ethnicity, and model 3 adjusting for age, sex, race/ethnicity, ALT, AST, education, smoking behavior, alcohol consumption, IPR, BMI, globulin, hypertension, diabetes, and stroke. Additionally, the relationship between albumin levels and DR impairment was evaluated using smooth curve fittings and generalized additive models. Subgroup analyses were conducted by stratifying for sex, age, and race/ethnicity, without adjusting for sex, age, and race/ethnicity in the models. Statistical analyses were performed using R (http://www.R‐project.org) and EmpowerStats (http://www.empowerstats.com), with a significance level of *P* < 0.05.

## 3 RESULT

**FIGURE 2:**
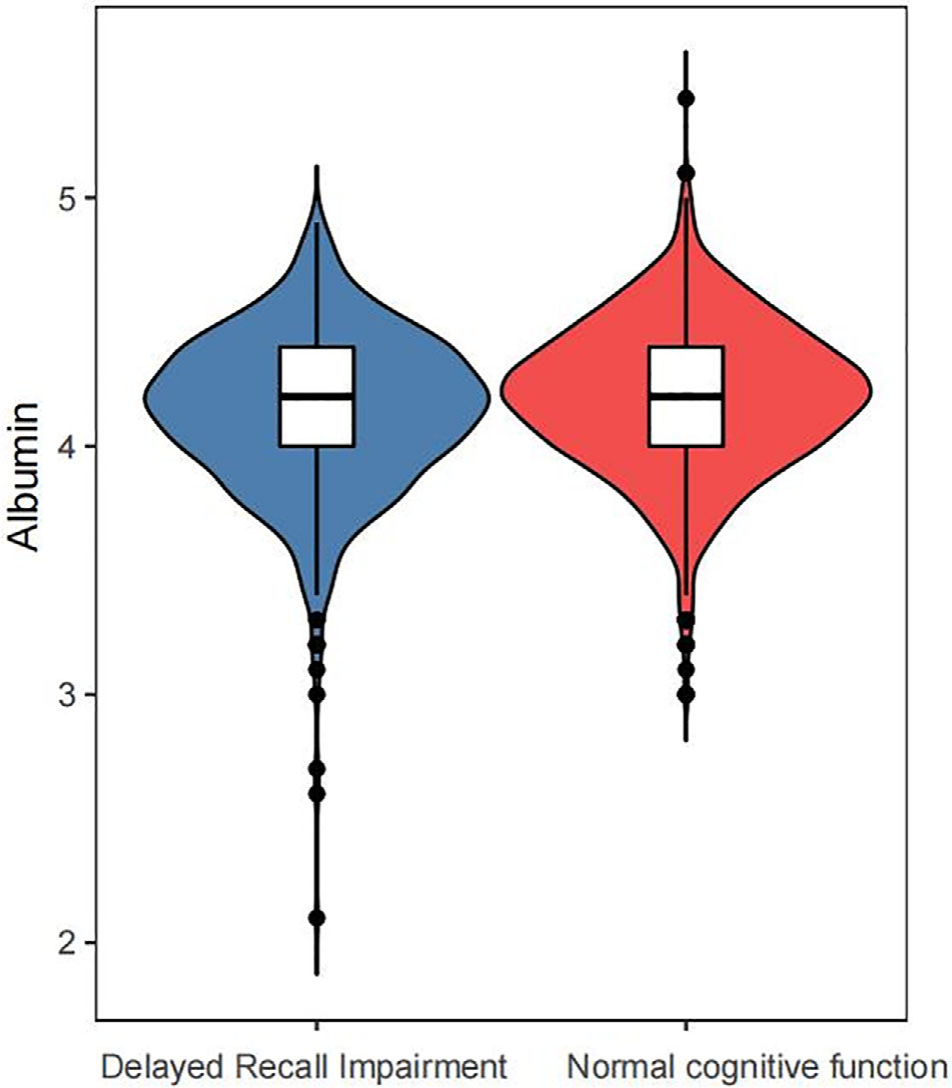
Distribution of albumin levels in cognitive function. Violin plot: blue violin represents delayed recall impairment, and red violin represents normal cognitive function.

A total of 2060 participants aged 60–80 years were included in the present study (Figure 1). Among them, 553 (26.8%) had cognitive impairment, whereas 1507 (73.2%) had normal cognitive function. Violin plots were used to illustrate the distribution of albumin levels in the two cognitive function groups (Figure 2).

Table 1 presents the basic characteristics of the study subjects. The cognitive impairment group exhibited a lower level of albumin (4.1 ± 0.3 g/dL) compared to the normal cognitive function group (4.2 ± 0.3 g/dL). Furthermore, participants with cognitive impairment were more likely to be male, Black, older, have lower education levels, and have poor income ratios. Additionally, a higher prevalence of diabetes, hypertension, and stroke was observed in the cognitive impairment group.

**TABLE 1:** Characteristics of participants according to cognitive function.

Variables	Normal cognitive function (*N* = 1507)	Delayed recall impairment (*N* = 553)	*p*‐Value
Age (year)	68.09 ± 6.33	72.18 ± 6.94	<0.001
BMI (kg/m^2^)	29.47 ± 6.60	28.56 ± 6.00	0.005
Albumin (g/dL)	4.20 ± 0.30	4.14 ± 0.33	<0.001
ALT (u/L)	22.42 ± 12.40	21.34 ± 13.91	0.092
AST (u/L)	25.21 ± 11.40	25.53 ± 14.31	0.604
Total protein (g/dL)	7.07 ± 0.49	7.06 ± 0.48	0.536
Globulin (g/dL)	2.88 ± 0.49	2.92 ± 0.50	0.046
Sex			<0.001
Men	671 (44.5%)	321 (58.0%)	
Women	836 (55.5%)	232 (42.0%)	
Race/ethnicity			0.452
Mexican American	154 (10.2%)	63 (11.4%)	
Other races	333 (22.1%)	114 (20.6%)	
Non‐Hispanic White	657 (43.6%)	228 (41.2%)	
Non‐Hispanic Black	363 (24.1%)	148 (26.8%)	
Level of education			<0.001
Less than high school	357 (23.7%)	224 (40.5%)	
High school	364 (24.2%)	141 (25.5%)	
More than high school	786 (52.2%)	188 (34.0%)	
Income to poverty ratio			<0.001
0–1.99	632 (41.9%)	303 (54.8%)	
1.99–3.49	314 (20.8%)	92 (16.6%)	
>3.49	438 (29.1%)	114 (20.6%)	
Missing	123 (8.2%)	44 (8.0%)	
Hypertension			0.212
No	585 (38.8%)	198 (35.8%)	
Yes	922 (61.2%)	355 (64.2%)	
Diabetes			0.039
No	1177 (78.1%)	408 (73.8%)	
Yes	330 (21.9%)	145 (26.2%)	
Stroke			<0.001
No	1418 (94.1%)	493 (89.2%)	
Yes	89 (5.9%)	60 (10.8%)	
Smoke at least 100 cigarettes in life			0.344
No	763 (50.6%)	293 (53.0%)	
Yes	744 (49.4%)	260 (47.0%)	
At least 12 alcohol drinks/1 year			0.005
No	497 (33.0%)	219 (39.6%)	
Yes	1010 (67.0%)	334 (60.4%)	

*Note*: Mean ± SD for continuous variables and percentage (%) for categorical variables.

Abbreviations: ALT, alanine aminotransferase; AST, aspartate transaminase; BMI, body mass index.

The results of the multivariate regression analyses are presented in Table 2. In the unadjusted model, albumin was found to be negatively correlated with cognitive impairment (OR: 0.52, 95%CI: 0.38–0.72, *P* < 0.05). This negative association persisted even after adjusting for confounders in model 2 (OR: 0.61, 95%CI: 0.44–0.86, *P* < 0.05) and model 3 (OR: 0.60, 95%CI: 0.41–0.87, *P* < 0.05). When albumin was converted from a continuous variable to a categorical variable (quartiles), individuals in the highest quartile (Q4) had a 35% lower risk of cognitive impairment compared to those in the lowest albumin quartile (Q1). This trend remained statistically significant across different albumin quartile groups (*P* < 0.05).

**TABLE 2:** The association between albumin (g/dL) and delayed recall impairment.

	Model 1 OR (95% CI) *p* value	Model 2 OR (95% CI) *p* value	Model 3 OR (95% CI) *p* value
Albumin (g/dL)	0.52 (0.38, 0.72) <0.001	0.61 (0.44, 0.86) 0.004	0.60 (0.41, 0.87) 0.007
Albumin categories			
Q1 (2.1–3.8)	Reference	Reference	Reference
Q2 (3.9–4.0)	0.69 (0.49, 0.96) 0.030	0.74 (0.51, 1.06) 0.096	0.73 (0.50, 1.06) 0.094
Q3 (4.1–4.3)	0.60 (0.45, 0.81) <0.001	0.67 (0.49, 0.92) 0.013	0.69 (0.49, 0.97) 0.031
Q4 (4.4–5.4)	0.56 (0.41, 0.76) <0.001	0.64 (0.45, 0.89) 0.008	0.65 (0.45, 0.94) 0.024
P for trend	<0.001	0.011	0.042

*Note*: Model 1: no covariates were adjusted. Model 2: age, sex, and race/ethnicity were adjusted. Model 3: age, sex, race/ethnicity, ALT, AST, education, smoking behavior, alcohol consumption, income poverty ratio, BMI, globulin, hypertension, diabetes, and stroke were adjusted.

Abbreviations: ALT, alanine aminotransferase; AST, aspartate transaminase; BMI, body mass index; CI, confidence interval; OR, odds ratio; Q1, quartile 1; Q2, quartile 2; Q3, quartile 3; Q4, quartile 4.

**FIGURE 3:**
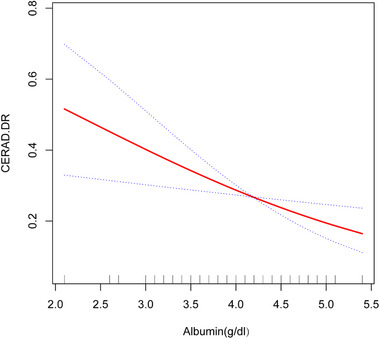
The association between albumin (g/dL) and risk of delayed recall impairment. Solid redline represents the smooth curve fit between variables. Blue bands represent the 95% of confidence interval from the fit. A linear relationship between them was detected after adjusting for age, sex, race/ethnicity, alanine aminotransferase (ALT), aspartate aminotransferase (AST), education, smoking behavior, alcohol consumption, income poverty ratio, body mass index (BMI), globulin, hypertension, diabetes, and stroke.

Furthermore, we employed generalized additive models and smooth curve fitting to assess linearity and validate the results. After adjusting for relevant covariates, a linear relationship between albumin levels (g/dL) and the risk of DR impairment was observed (Figure 3).

**FIGURE 4:**
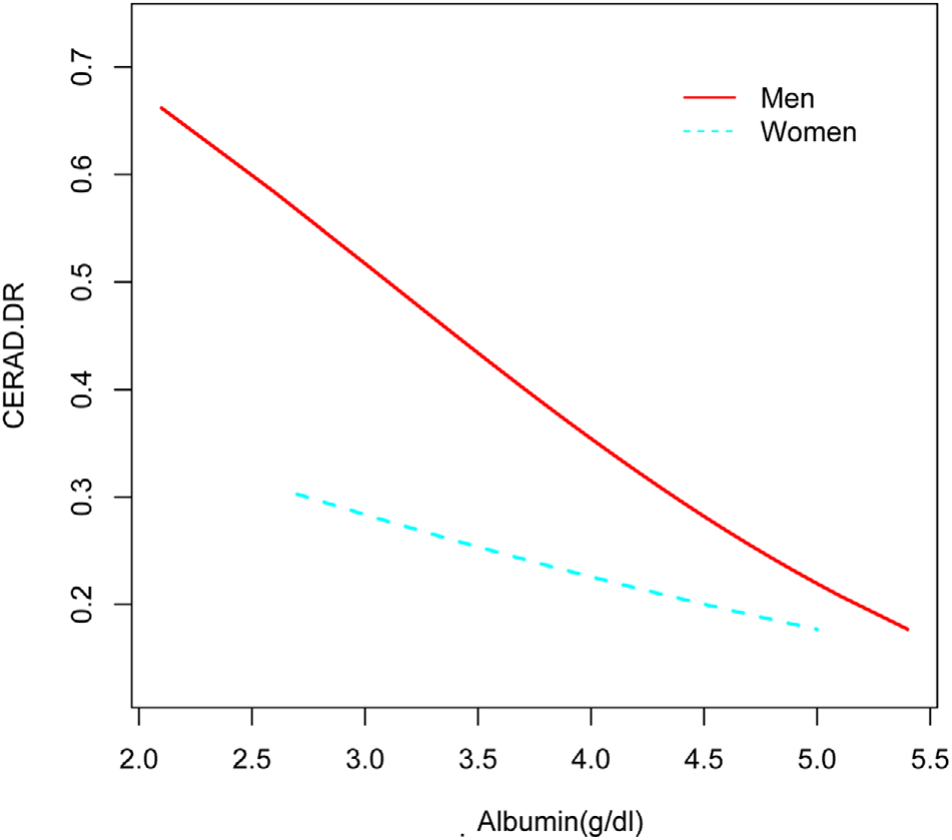
The association between albumin (g/dL) and risk of delayed recall impairment is stratified by sex. A linear relationship between them was detected after adjusting for age, race/ethnicity, alanine aminotransferase (ALT), aspartate aminotransferase (AST), education, smoking behavior, alcohol consumption, income poverty ratio, body mass index (BMI), globulin, hypertension, diabetes, and stroke.

Stratified by sex, a similar linear relationship was observed after adjusting for the same covariates (Figure 4).

**FIGURE 5:**
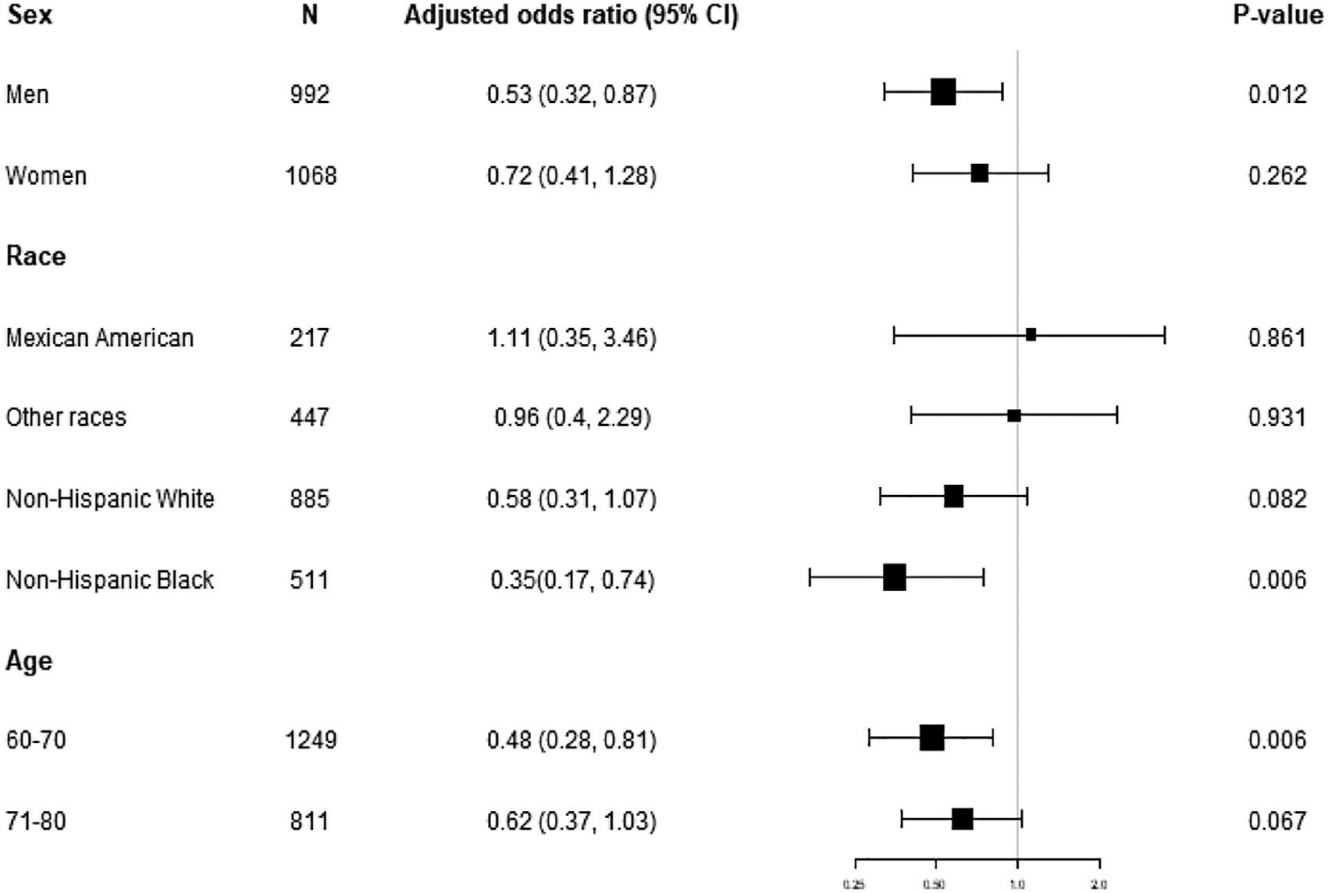
Forest plots: The association between albumin (g/dL) and the risk of delayed recall impairment is stratified by sex, race/ethnicity, and age; the model is not adjusted for sex, race/ethnicity, and age, respectively. Alanine aminotransferase (ALT), aspartate aminotransferase (AST), education, smoking behavior, alcohol consumption, income poverty ratio, body mass index (BMI), globulin, hypertension, diabetes, and stroke were adjusted.

Subgroup analyses stratified by sex, race, and age using a forest plot demonstrated that the fully adjusted regression model exhibited a negative correlation in men (OR: 0.53, 95%CI: 0.32–0.87, *P* < 0.05), Blacks (OR: 0.35, 95%CI: 0.17–0.74), and individuals aged 60–70 years (OR: 0.48, 95%CI: 0.28–0.81), but not in women, Whites, Mexican Americans, or those aged 71–80 years (Figure 5).

## 4 DISCUSSION

The present study aimed to investigate the association between albumin levels and DR impairment in a nationally representative sample of older adults in the United States. Our findings reveal that higher albumin levels are significantly associated with a reduced risk of DR impairment, particularly among older men, individuals aged 60–70 years, and Blacks.

Several studies have previously reported an independent association between low serum levels of albumin and poor cognitive performance in older adults (Ng et al., 2008, 2009). In contrast, previous studies have reported no significant association between albumin levels and cognitive function, as assessed by various cognitive tests, including mini–mental state examination (MMSE), auditory verbal learning test, coding task, and Raven's colored progressive matrices, among both older adults with normal cognitive function and the general older population (Dik et al., 2005; Ravaglia et al., 2005). The inconsistent findings may be attributed to variations in the measures used to assess cognitive impairment as well as the heterogeneity of the populations studied. However, a recent nationally representative population‐based study conducted in England, which included 1752 adults aged 65 years and older from the Health Survey for England 2000, utilizing multilevel stratified sampling and multivariable logistic regression models, provided evidence that low serum albumin levels are independently associated with increased odds of cognitive impairment in the elderly population (Llewellyn et al., 2010). The Chinese Longitudinal Healthy Longevity Survey (CLHLS) investigated eight regions with high longevity. All subjects aged 65 years and older were recruited and assessed using the MMSE scale. The findings of the study revealed that higher levels of serum albumin were associated with a reduced risk of cognitive impairment (Yin et al., 2016). Furthermore, another study involving 1800 Chinese older adults aged 60 and above at baseline reported that lower serum albumin concentrations may serve as an independent risk factor for mild cognitive impairment in the elderly (Wang et al., 2018). Our findings are in line with previous studies, and we further strengthen the results by employing stratified analysis. Additionally, we extend the existing knowledge on the association between serum albumin levels and cognitive performance by utilizing smooth curve fitting to visually illustrate the significant relationship. Notably, our study differs from previous research as we employed the CERAD‐DR assessment tool to evaluate cognitive function.

There are several plausible mechanisms through which serum albumin may be associated with cognitive impairment in older adults. First, serum albumin has been linked to nutritional status (Covinsky et al., 2002). Persistent malnutrition has been shown to play a pivotal role in cognitive impairment (Karakis et al., 2016; Yildiz et al., 2015). Therefore, lower serum albumin levels may reflect malnourishment and potentially contribute to the progression of cognitive impairment. Second, serum albumin is the main component of maintaining plasma colloid osmotic pressure and blood volume (Ohlsson et al., 1981). Blood pressure and other vascular factors are also linked to cognitive impairment (Sabayan & Westendorp, 2015; Yuan et al., 2016). Consequently, reduced levels of albumin may interfere with blood supply to the central nervous system, potentially leading to cognitive impairment. Third, serum albumins have significant antioxidant activity (Guo et al., 2011; Ishizaka et al., 2007), and oxidative stress has been recognized as a significant factor in dementia (Mao, 2013; Owen et al., 1997). Decreased albumin levels could disrupt the oxidant/antioxidant balance and further contribute to cognitive impairment. Moreover, evidence suggests that serum albumin can bind to amyloid‐β, attenuating its neurotoxicity and preventing cognitive decline by reducing its aggregation (Stanyon & Viles, 2012).

In our study, we observed a significant association between albumin levels and DR impairment in older men, whereas no statistically significant difference was found in women. However, a weak trend was noted in women, with elevated albumin levels showing a potential protective effect against cognitive impairment (OR: .72, 95%CI: .41−1.28, *P* = .262). It is plausible that this observed trend in women may be attributed to the limited sample size of our study or the potential limitations of the cognitive function test utilized in this population. Furthermore, it should be noted that cognitive function tests alone may not accurately reflect the complex and multifactorial nature of Alzheimer's disease. Interestingly, in our study, we observed an inverse correlation between albumin levels and cognitive impairment, specifically in Blacks, but not in other racial/ethnic groups. This finding may be attributed to differences in diet patterns and genetic risk factors among different ethnic groups, which warrant further investigation. The racial/ethnic distribution in our study was as follows: whites accounted for 42.96%, Blacks for 24.81%, Mexican Americans for 10.53%, and other races for 21.7%. However, due to the relatively small sample size in some ethnic groups, the observed difference was not statistically significant. Furthermore, our findings suggest that the risk of DR impairment gradually decreased as albumin levels increased in individuals aged 60–70 years, but not in those older than 70 years. We hypothesize that the influence of confounding factors may be more pronounced in individuals over 70 years of age. Therefore, early screening and intervention for DR impairment may be particularly critical in individuals aged 60 and above.

This study used the utilization of a large nationally representative sample of adults in the US. The data showed credibility and repeatability. To our knowledge, this study is the first to report the relationship between DR impairment and albumin levels among Blacks. However, this study had several limitations. First, the cross‐sectional study does not allow us to establish causality. Further prospective randomized controlled trials are needed to validate these results. Second, participants in this study were recruited only from the United States. The results may not be generalized to other countries. Third, our study did not fully consider other health conditions affecting albumin levels such as kidney disease, and these factors will need to be considered in future studies. Fourth, our subgroup analyses and their results are exploratory as these are not established a priori.

Patients with impaired cognitive function may exhibit suboptimal dietary patterns or reduced awareness, which could potentially result in decreased dairy intake. Additionally, eating difficulties are commonly observed in individuals with advanced dementia, often leading to malnutrition (Mitchell et al., 2009). Malnutrition in dementia has detrimental consequences, including increased morbidity and mortality, diminished quality of life, and heightened caregiver burden (Avila et al., 2004; Faxén‐Irving et al., 2005). Therefore, addressing and correcting malnutrition may be crucial for improving the prognosis of patients with dementia.

## 5 CONCLUSION

Our study suggested elevated albumin levels correlated with decreased risk of DR impairment, especially in older men and Blacks. Thus, older patients with low albumin need timely cognitive assessment and intervention for cognitive decline. This finding indicated that maintaining albumin at high levels may be beneficial for the cognitive function of older adults.

## AUTHOR CONTRIBUTIONS

Y Hu, H Luo and G Li conceived and designed the study. D Lin, M Song, and D Wu conducted the formal analysis. Y Zhang developed the methodology. Y Hu wrote the initial drafts. D Lin and M Song helped draft the manuscript. G Li and H Luo are the corresponding authors of this work and supervised work on the entire manuscript. All the authors read and approved the final manuscript.

## CONFLICT OF INTEREST STATEMENT

The authors have no conflicts of interest to report.

## FUNDING INFORMATION

This research was funded by the Natural Science Foundation of Chongqing (CSTB2023NSCQ‐MSX0176).

### PEER REVIEW

The peer review history for this article is available at https://publons.com/publon/10.1002/brb3.3435.

## Data Availability

The survey data are publicly available on the internet for data users and researchers throughout the world (www.cdc.gov/nchs/nhanes/).
